# Syphilology

**Published:** 1876-01

**Authors:** 


### IV. Syphilology.

1. *Transmission of Syphilis between Nurses and Nurslings.* Taylor. (*Am. Jour. of Obst. and Diseases of Women and Children*, Nov.,'75.

This paper is written in the lucid and practical manner, which is an admirable feature of the style of the author. The distinction between specific and other lesions of the mouth in infants is thus given : In syphilis, the coryza, which most constantly accompanies the mouth lesion, is much more severe, and the snuffling much greater, and there will be observed a tendency for the lesions to develop at the angles of the mouth, and there to induce ulceration, which may extend to the integument. This is important. In syphilis, the focus of the inflammation is developed upon the tongue, the fauces and the region named ; whereas, in the simple form of sore mouth, the tissues generally, except the throat, are involved and the gums are affected, while they most constantly escape in syphilis. Again, in the simple form of trouble, the sulci between the lips and the teeth are often implicated, and in syphilis it is usual for them to escape.

Taylor also calls attention to two important practical points: 1. The danger of mediate contagion, in children's hospitals and asylums, from the use of an artificial nipple upon which the secretion from the mouth of a syphilitic infant might be retained ; and 2. The danger of pronouncing the breast of a nurse free from syphilitic lesions, so long as there is a possibility of the existence of a chancre *in the stage of incubation* upon or near the nipple.

Taylor's article should be placed in the hands of every physician in the country, who is connected with a children's asylum or hospital.

2. *Ulcerating Syphilide and Lupus Vulgaris.* Duhring. (*Med. and Surg. Reporter*, December 4.)

The distinction between the two diseases is thus made, in a clinical study of a patient affected with the first named disorder:

The recognition of the non-inflammatory character of the lesion, excludes a large class of diseases in diagnosis. It is a neoplasm, and must be due either to lupus vulgaris or syphilis. Were it lupus, we should expect to see some signs of this deposit in the form of papules and tubercles, which are wanting. There would be also dry, flaky desquamation; but the skin is free from scales. The infiltration would not be uniformly distributed (as it is); it would manifest a broken appearance, the sound skin showing between the smaller patches which always go to make up the larger tracts of lupus. On the other hand, the uniform infiltration, the elevation of the patch, the appearance of the ulcers and their tendency to communicate subcutaneously, the distinct semi-circular lines of demarcation surrounding the patch upon the side of the head, the absence of pain, and other symptoms, all point to a late syphilide. Amelioration and aggravation of the condition have been undoubtedly produced by repeated courses of iodide of potassium. Duhring recommended the use of the latter remedy, in combination with mercury and dilute mercurial ointment locally.

3. *Laryngeal Mucous Patches.* ISAMBERT. (*Le Progrès Médical*, Oct. 9.).

The name should be exclusively applied to lesions which have morphological characters identical with mucous patches of the genitals, mouth or velum. They should be irregularly round or oval; should project slightly above the surrounding tissue; should be circumscribed by a border a little more salient than the centre, which is depressed. The surface is of a grayish-opaline hue, is delicately folded or swollen, and surrounded, in recent lesions, by a more or less intensely defined inflammatory zone, of a brownish hue at or adjacent to the external integument, carmine-colored, when resting upon an internal mucous surface. It rapidly loses these features when it ulcerates, and, in order to be fully recognized, should at least exhibit the elevated rim, the folded aspect and the opaline shade. The lecturer agreed with Fournier, Duploy and Ferras, who consider that this typical form is rarely encountered in the larynx: and adds that in exceptional cases it has been noted where there was no specific diathesis.

The constant temperature and continual humidity of the expired air observed in the larynx, render its lesions analogous to those of the skin, which are subjected to continued cataplasms or indefinitely prolonged vapor-baths. Hence the rarity of recognition of typical mucous patches in this region. Subjected to these conditions their features are altered. The most marked of the cases observed, have been occasioned by coincident laryngeal syphilis and phthisis.

4. *Syphilitic Laryngeal Vegetations and Pulmonary Gummata.* MAUNOIR. (*Le Progrès Médical*, Oct. 9.)

A laundress, 40 years old, had cutaneous syphilides, aphonia, dyspnœa requiring tracheotomy, and pulmonary symptoms, resulting fatally.

The inferior vocal cords were thickened, but not ulcerated. Above them were three or four polypiform, reddish vegetations, nearly as large as peas, which projected beyond the free border of each vocal cord. (Hence the sinuous chink observed, ante mortem, by the laryngoscope.) The tracheo-bronchial membrane was reddened, and smeared with viscid adherent mucus.

Small, rounded, yellowish-white tumors, with a fibrous zone at the periphery, were found at the summits of the lungs—especially the right—imbedded in sound pulmonary tissue. Open-mouthed vessels and bronchioles were visible in the centre of each. Some were soft and caseous. One was connected with a small vomica. Some were firm, opaline and semi-transparent. They differed from tubercles, in their definite outlines, their intact pulmonary bed, and the patent central canals—vascular and bronchial.

5. *Syphilitic Liver.* THOMPSON, HENRY. (*Lancet*, November, 1875.)

Post mortem, the viscus was found to be displaced downwards; bands of adhesion united it to the parietal peritoneum, all vascular, the vessels running from the liver to the abdominal wall. The hepatic lobes were broken up by deep furrows into a multitude of irregular knobs, the entire surface being more or less granular. The capsule presented unevenly distributed thickenings. There was also well marked fibrous thickening at the bottom of the furrows, and along the course of the larger portal canals; and here and there were seen small tracts of caseous character in the midst of the denser material. The liver substance throughout was permeated and toughened by this fibrous change, so that it was nearly impossible to distinguish any individual lobules—the general appearance being that of fine granules of various sizes and shades of color, interspersed with large masses of a dark-green tint. The organ weighed 56½ oz. There was no dilatation of the interstitial bile ducts; in fact, their presence could hardly be ascertained. The hepatic and common bile ducts were also free.

Iodide of potassium utterly failed. Sir Henry Thompson adds, with great wisdom, that when the characteristic changes in the liver are well pronounced, iodide of potassium will no more undo the mischief than it will abolish a cicatrix.

6. *Rapidly Malignant Syphilis.* GUIBOUT. (*L' Union Méd.*, 61, 62, 1875.)

Syphilitic rupia with continuous fever, great prostration and weakness, serious functional disorders, and extensive ulceration, all occurred within six weeks of the primary lesion, without the intervention of the ordinary milder syphilides.

These cases are sufficiently common to require attention. They usually result from unrecognized or mal-treated chancre.

7. *Syphilitic Placenta.* MACDONALD. (*Obst. Jour. of Great Britain and Ireland*, Oct.)

It may be mistaken for fatty degeneration (Kilian and Robin), but it is easy to recognize constitutional syphilis in a dead-born fœtus, even if macerated. There is a band of tissue between the bone of the shaft and the cartilage of the epiphysis of the long bone, in a condition of inflammatory irritation. It is bounded by very irregular outlines, both towards the cartilage and towards the true bone, and consists, according to the advancement of the lesion, either of (*a*) hypertrophied cartilaginous cells, greatly increased by proliferation and prematurely infiltrated with earthy matter; or (*b*) of the same, combined with premature sclerosis of the intercellular tissue, and premature osteogenic formation within the cartilage, and arrest of true bony transformation; or (*c*) of the higher degrees of inflammation, softening and interrupting the connection between bone and cartilage, with inflammatory exudation, or even suppuration. The red or grayish-yellow band is readily seen, and the hardened prolongation of premature calcified cartilage easily felt and seen.

In the placenta, if the father is primarily affected, there is cellular hypertrophy and multiplication of the villi ("Disfiguring granulation-cell disease"—Frænkel), involving contents, epithelial mantle, and wall of included vessel, proceeding outward from the vessels, the rows of connective tissue nuclei suggesting Haversian canals. In consequence of the accompanying increase in size and weight, the vascular destruction and hypertrophy are soon followed by atrophy and abortion of the villus. Congestion and extravasation are likely to attack the unaffected portions of the placenta, and suffocation of the fœtus ultimately results.

If the mother is primarily affected, there is increased growth of the connective tissue framework of the maternal placental decidua, with enormous hypertrophy of its large cells, leading to destruction of the villi by compression. The endometritis and placentaris nummosa of Virchow, Slavjansky, and Kleinwächter, are probably syphilis of the maternal placenta.

If both parents are primarily syphilitic, or become so early in pregnancy, the conditions described above occur conjointly.

8. *Syphilis of the Placenta.* FITZ. (*Boston Med. and Surg. Jour.*, Dec., 1875.)

The placenta exhibited a thickened and opaque uterine surface, which presented yellowish patches, alternating with others of a grayish-white color. Beneath these were nodular masses, corresponding in color, and of a relatively homogeneous appearance, the yellow nodules being more dense than the gray ones. The membranes were also thickened, opaque in spots, and of a yellowish color. The changes in the placenta were due to a thickening and increased formation of the placental villi, with cell formation and an eventual fatty degeneration.

These changes indicate paternal infection of ovum, without maternal syphilis (Frænkel). The bones of the fœtus (five months old, and macerated) confirmed the diagnosis. There was no sharp dividing line between the bone and the cartilage. There had been frequent previous abortions and probable syphilis of father.

9. *Prognosis in Syphilis.* HUTCHINSON. (*Lancet.*)

The author calls attention to a fact which so many are slow to appreciate. viz., that it is impossible to assure a patient that he has not contracted syphilis, until at least a month has elapsed after suspicious intercourse. It must be remembered that induration may not occur for weeks. If contagion were effected with as much care as to purity of the virus as is exercised in selecting lymph for vaccination, then the result might be confidently predicted. It is, however, not so; and the introduction of pus

(chancrous or other) with the infecting element, influences the results, immediate and deferred.

There is nothing new in all this, but the profession cannot be too often warned of possible errors on this point.

10. *Treatment of Primary Syphilis by Lewin's Method.* SEELEY. (*Nashville Jour. of Med. and Surg.*, Oct.)

The patient had well marked indurated chancre. From the 3rd to the 28th of May, S. injected hypodermically, with tolerable regularity, ten drops of Lewin's solution of corrosive sublimate. There was excessive gastric irritability (relieved by rest, ice to the spine and mustard cataplasms), and the characteristic indurations at the site where the needle entered. Cure was apparently perfect.

11. *Efficacy of Iodide of Potassium in Syphilis.* MCSWEENY. (*British Med. Jour.*, Sept.)

Five grains of the potassic iodide, combined with three grains of carbonate of ammonia, are as efficient as eight grains of the potassium salt administered in the ordinary way.

12. *Treatment of Phagedenic, Gangrenous, Venereal Sores.* SIMMONS. (*Med. Record*, Sept. 11.)

The diseased parts are immersed continuously in hot or warm water. In one case, where there had been loss of labia, fourchette, clitoris, and part of the urethra, the fetid pus from the immense irregular cavity became laudable after thirty-six hours' use of the sitz-bath and cushion. Relief was so great that the patient refused to leave the bath longer than was actually necessary. After the desired change was produced, the applications were made only during alternate hours.

Several cases, with equally favorable results, are reported.

13. *Treatment of Papulo-hypertrophic Syphilides.* CHÉRON. (*La France Médicale*, Nov. 10.)

These lesions are painted with a solution of nitrate of silver (fifty per cent. strength), and then touched with the zinc crayon, the operation being repeated every second or third day. Nine days usually suffice for complete removal.

14. *Tayuya—a new Antisyphilitic.* UBICINI. (*Bulletin Gén. de Thérapeutique*, Sept.)

The remedy was found to be efficacious among the Brazilian negroes. Martin discovered in a specimen: green resin; some yellow, unctuous matter; a very bitter, highly aromatic brown extractive; tannin; starch; mucilage; volatile matter; magnesia; alumina; lime; iron; potash and woody matter. The mineral substances were exceedingly abundant, and precipitated at once from a strong solution.

15. *Syphilis Variously Treated in Pregnancy.* WEBER. (*Am. Jour. of Med. Sciences*, Oct.)

(*A.*) Interference with the digestive tract predisposes to untimely birth. (*B.*) Exclusively local treatment resulted in 20 per cent. of premature labors. (*C.*) Local treatment, combined with inunction, left the pregnancy undisturbed. (*D.*) Inunction, accompanied or followed by iodine, was less satisfactory. (*E.*) Treatment by iodide of potassium and corrosive sublimate, was attended by 15 per cent. of premature births—in cases where the first named remedy alone was employed, 42 per cent. (*F.*) The long duration of treatment was less unfavorable than irritation of the intestinal tract. (*G.*) The period of commencing general treatment does not appear to influence the occurrence of premature births. (*H.*) The stage of development of syphilis does seem to exercise such an influence.

These deductions are based upon observation of 129 pregnant women, suffering from syphilis.

